# A framework for extending trial design to facilitate missing data sensitivity analyses

**DOI:** 10.1186/s12874-020-00930-2

**Published:** 2020-03-17

**Authors:** Alexina J. Mason, Richard D. Grieve, Alvin Richards-Belle, Paul R. Mouncey, David A. Harrison, James R. Carpenter

**Affiliations:** 1 Department of Health Services Research and Policy, London School of Hygiene and Tropical Medicine, 15-17 Tavistock Place, London, WC1H 9SH UK; 2 Clinical Trials Unit, Intensive Care National Audit & Research Centre (ICNARC), London, UK; 3 Department of Medical Statistics, London School of Hygiene and Tropical Medicine, Keppel Street, London, WC1E 7HT UK; 4 MRC Clinical Trials Unit at UCL, 90 High Holborn, London, WC1V 6LJ UK

**Keywords:** Bayesian analysis, Clinical trials, Expert elicitation, Missing data, Pattern-mixture models, Sensitivity analysis

## Abstract

**Background:**

Missing data are an inevitable challenge in Randomised Controlled Trials (RCTs), particularly those with Patient Reported Outcome Measures. Methodological guidance suggests that to avoid incorrect conclusions, studies should undertake sensitivity analyses which recognise that data may be ‘missing not at random’ (MNAR). A recommended approach is to elicit expert opinion about the likely outcome differences for those with missing versus observed data. However, few published trials plan and undertake these elicitation exercises, and so lack the external information required for these sensitivity analyses. The aim of this paper is to provide a framework that anticipates and allows for MNAR data in the design and analysis of clinical trials.

**Methods:**

We developed a framework for performing and using expert elicitation to frame sensitivity analysis in RCTs with missing outcome data. The framework includes the following steps: first defining the scope of the elicitation exercise, second developing the elicitation tool, third eliciting expert opinion about the missing outcomes, fourth evaluating the elicitation results, and fifth analysing the trial data. We provide guidance on key practical challenges that arise when adopting this approach in trials: the criteria for identifying relevant experts, the outcome scale for presenting data to experts, the appropriate representation of expert opinion, and the evaluation of the elicitation results.The framework was developed within the POPPI trial, which investigated whether a preventive, complex psychological intervention, commenced early in ICU, would reduce the development of patient-reported post-traumatic stress disorder symptom severity, and improve health-related quality of life. We illustrate the key aspects of the proposed framework using the POPPI trial.

**Results:**

For the POPPI trial, 113 experts were identified with potentially suitable knowledge and asked to participate in the elicitation exercise. The 113 experts provided 59 usable elicitation questionnaires. The sensitivity analysis found that the results from the primary analysis were robust to alternative MNAR mechanisms.

**Conclusions:**

Future studies can adopt this framework to embed expert elicitation within the design of clinical trials. This will provide the information required for MNAR sensitivity analyses that examine the robustness of the trial conclusions to alternative, but realistic assumptions about the missing data.

## Background

Randomised controlled trials (RCTs) typically deal with missing outcome data using analysis methods that assume the outcomes are ‘missing at random’ (MAR). However, for patient-reported outcome measures (PROMs), this assumption is often implausible. This is because the reasons that patients are lost to follow-up, or do not complete the requisite questionnaires, are related to their unobserved health status (even after taking account of observed data). This can bias estimates of effectiveness, underestimate uncertainty, and cause those interpreting the results to draw incorrect conclusions [1–3].

The trial design should attempt to minimise levels of missing data, but where missing data are inevitable, appropriate analysis strategies should be developed at the design stage [4]. Methodological guidelines recommend that studies undertake sensitivity analyses which recognise that such data may be ‘missing not at random’ (MNAR) [4, 5]. These sensitivity analyses may usefully be informed by expert opinion about the likely average differences between observed and missing outcomes. Previous approaches to elicitation for MNAR sensitivity analysis in trials and epidemiology relied on paper questionnaires or spreadsheets [6, 7]. More recently, we developed an easy-to-use web-based elicitation tool to quickly elicit expert opinion about patients with missing versus complete data [8]. However, these studies were implemented post hoc and not incorporated within the RCT design.

For trials to routinely undertake appropriate MNAR sensitivity analyses informed by expert opinion, key challenges have to be tackled in the design and use of the elicitation exercise. In his critique of our previously published elicitation tool, Heitjan[9] raised the crucial question: do the experts understand the questions? This raises issues about the selection of experts, the presentation of the outcome scales, the representation of the expert views, and the assessment and use of the elicitation results. We develop a framework for elicitation that can be directly incorporated in the main trial design to directly address these important concerns, taking a Bayesian perspective that can naturally combine expert opinion with the current trial data through its prior distributions [10].

In this paper, and consistent with the primary trial report [11], our focus is on the ITT estimand. Note that, because the intervention concluded before the outcome was measured, this is not a hypothetical estimand (in the sense of the ICH E9 addendum [12]).

Our proposed elicitation framework was developed for the cluster-randomised POPPI trial (details next section). The framework includes guidance on dealing with four key challenges:

1. Identification of experts
2. Presentation of the outcome scales: how do we make these interpretable by non-statistical experts?
3. Representation of expert opinion: what is the best balance between flexibility and ease of use when eliciting mean values?
4. Assessment of elicitation results: how much confidence should we have in the elicited responses?

We advocate that the elicitation exercise and MNAR sensitivity analysis are described in the trial’s statistical analysis plan (SAP) and carried out concurrently with the main analysis.

The remainder of the article proceeds as follows. Next we introduce the POPPI trial, followed by presentation of our framework, illustrative results from the elicitation exercise and associated sensitivity analysis. We conclude with a discussion of the strengths and challenges of elicitation, and outline areas for further research.

## Methods

### The POPPI trial

The Psychological Outcomes following a nurse-led Preventive Psychological Intervention for critically ill patients (POPPI) trial was a multi-centre, parallel-group, cluster-randomised trial, comparing a nurse-led preventive, complex psychological intervention, initiated in the ICU, with usual care (ISRCTN53448131, registered 16/7/2015) [13]. The intervention comprised promotion of a therapeutic ICU environment, three stress support sessions and a relaxation and recovery program (see Supplement for further detail). Randomisation was at the ICU level. The primary clinical effectiveness outcome is mean difference in patient-reported PTSD symptom severity at six months between patients receiving the POPPI intervention versus usual care. Patient-reported PTSD symptom severity is measured using the PTSD Symptom Scale - Self Report questionnaire (PSS-SR) [14], with range 0 to 51. The health-related quality of life (HrQoL), measured using the EQ-5D-5L self-reported questionnaire [15], at six months, is on a scale anchored at -0.28 and 1 (for our elicitation exercise we multiply by 100 to ease completion).

POPPI recruited 1458 patients following admission to one of 24 intensive care units (ICUs) in the United Kingdom. Loss to follow-up was anticipated to be 20% among survivors. For the primary analysis, the SAP specified multiple imputation to handle the resulting missing outcome data assuming MAR. However, the outcomes were plausibly MNAR so the SAP specified a MNAR sensitivity analysis informed by expert opinion [16].

From the primary analysis, assuming MAR, the clinical effectiveness estimates were −0.03 (95% confidence interval −2.58 to 2.52; *p*=0.98) for PSS-SR and 0.007 (95% CI −0.063 to 0.076; *p*=0.85) for HrQoL. As anticipated, 79% and 77% of patients who survived to 6 months returned completed PSS-SR and EQ-5D-5L questionnaires respectively. The robustness of these results to alternative MNAR assumptions was reported in the primary paper [11].

### Proposed framework

**Fig. 1:**
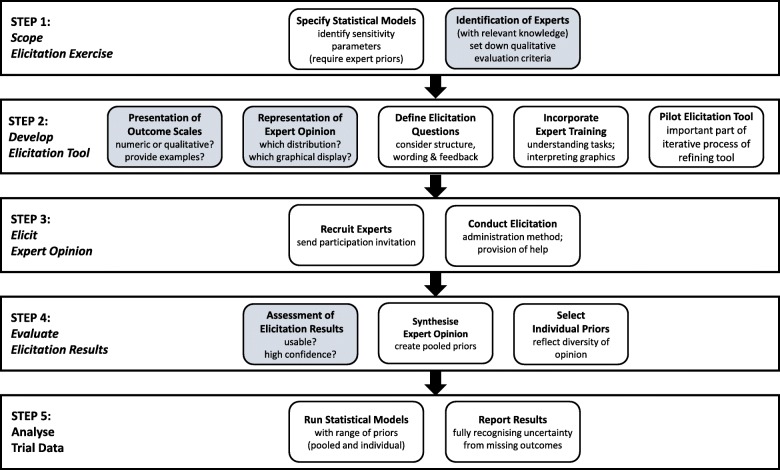
Expert elicitation framework. Elements shaded grey relate to the four challenges discussed in the introduction

Figure 1 shows the key elements in our framework for eliciting expert priors for a MNAR outcome sensitivity analysis in clinical trials, grouped into five steps. We assume that the primary MAR analysis model has been specified. The first step is to specify how the primary analysis model is generalised to allow outcomes to be MNAR. In particular, we need to identify the additional parameters which require expert priors. Steps 2–4 outline the elicitation exercise, which provides the expert priors. In the final step, the statistical models are run using these priors.

### Step 1: scope elicitation exercise

#### Specify statistical models:

Our approach to modelling MNAR outcomes is pattern mixture modelling, in which – within appropriate subgroups – elicited parameters specify the differences between fully observed, and partially observed, outcome data [5]. For POPPI we specified a pattern-mixture model that allows the mean PSS-SR (or HrQoL) score at six months to differ according to whether the scores are observed (pattern 1) or missing (pattern 2). For pattern 1, we can calculate the mean response for patients receiving usual care or the ‘POPPI intervention’ from the observed data. For pattern 2, the outcome data are missing, and so we calculate the mean to be that for pattern 1 plus an offset, termed *Δ*. Our Bayesian analysis estimates effectiveness of treatment by weighting the mean scores in each pattern by the proportion of patients with missing outcome scores. However, the distribution of *Δ* cannot be estimated from the observed data. For this type of model, the parameters specifying the distribution of *Δ*, and how this may vary with patient characteristics and intervention groups, are the sensitivity parameters which require expert priors (see Supplement).

To specify priors for the sensitivity parameters, our approach is to elicit expert opinion about the likely values of the difference in the mean outcomes between patients who did and did not complete the relevant questionnaire. This comparison should be made for patients who are similar according to observed characteristics. These variables are those anticipated to predict the missing data and the outcome, and will generally include age and gender. For POPPI, we also included the patient’s level of anxiety after regaining capacity in the ICU. The sensitivity parameter should also be allowed to differ by randomised arm, and so in POPPI we elicited the required values for patients according to whether they received usual care or the POPPI intervention.

#### Identification of experts:

A pool of experts with relevant knowledge must be identified at the outset. Here an expert is defined as having knowledge about why the outcome might be missing as well as the likely determinants of patients’ outcome at 6 months post ICU admission, according to whether or not they received the POPPI intervention. The qualitative evaluation criteria that will be used to assess the elicitation results should also be defined in advance. The elicitation tool should be prepared taking account of the experts’ likely experience and knowledge, the evaluation criteria and the method of administration, since these have implications for the level of numeracy assumed, questions asked, language used and the training element.

### Step 2: develop elicitation tool

The elicitation tool should be quick and easy to administer, and the questions should be accessible. A graphical approach can help experts represent their views intuitively, and naturally produces prior distributions for statistical models. Our POPPI elicitation tools used this method and were developed using Shiny, a web application framework within the statistical software, R [17, 18]. We now discuss the five important elements in the preparation of the elicitation tool which are summarised in Fig. 1, step 2.

#### Presentation of outcome scales:

**Fig. 2:**
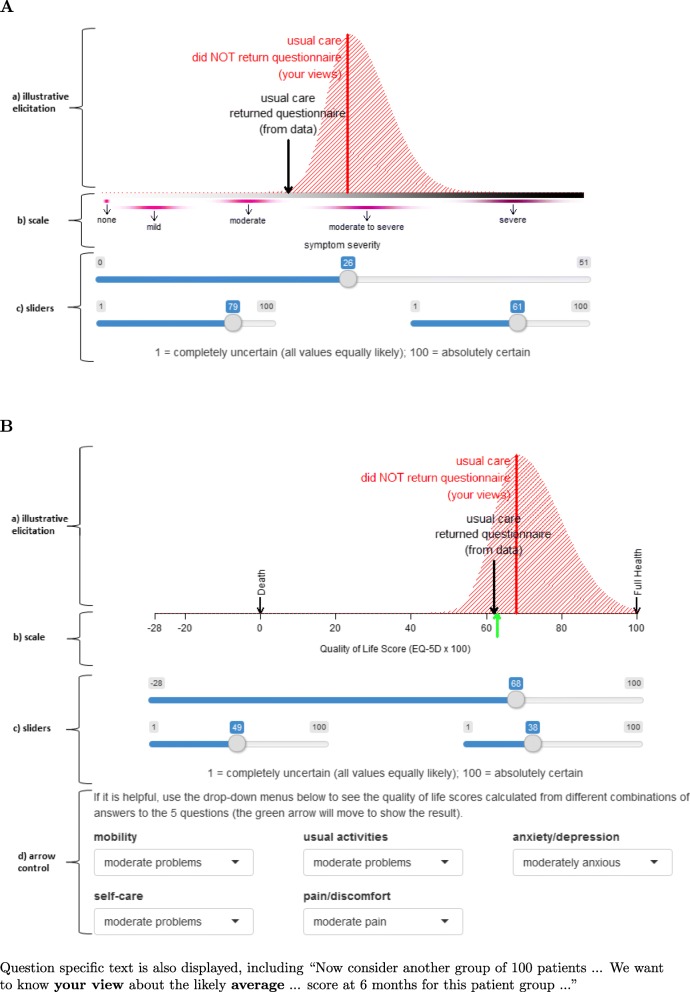
Screen shots showing the outcome scales from the two POPPI Elicitation Tools. **A** outcome scale for PSS-SR (PTSD symptom severity) scores. **B** outcome scale for EQ-5D-5L (HrQoL) scores

We must consider whether the experts will be able to easily associate the numeric outcome scores with the patients’ health status. If not, then the scales can be customised using descriptive labels and/or examples. Figure 2 illustrates different approaches for a unidimensional (PSS-SR) and a multidimensional (HrQoL) outcome. Panel A is a screen shot from the PSS-SR elicitation tool, with three regions labelled ‘a: illustrative elicitation’, ‘b: scale’ and ‘c: sliders’. The screen shot from the HrQoL elicitation tool (Panel B) shows a fourth region, ‘d: arrow control’.

#### Symptom severity scale:

PSS-SR (PTSD symptom severity) scores can take values from 0 to 51, with 0 indicating no symptoms and higher scores associated with a worse outcome. To ensure the scale was meaningful to our experts, we marked 0 as ‘none’ and placed a continuum of categories below the scale axis of the graph (Fig. 2, panel A, region b). The ranges associated with each category of symptoms were drawn from published literature: ‘mild’ (1-10), ‘moderate’ (11-20), ‘moderate to severe’ (21-35) and ‘severe’ (36-51).

Figure 2, panel A, region a, shows illustrative results from an elicitation. The vertical arrow shows the average PSS-SR score (calculated from a sample of patients recruited early to the study) for the sub-group of patients who received usual care and returned their questionnaires. The shaded curve (distribution) above the scale indicates that the expert believes that the average PSS-SR scores for this sub-group of patients who received usual care and did not return a completed questionnaire, lies between the ‘moderate’ and the ‘severe’ symptom severity scores.

Panel A, region c, shows the sliders which the expert uses to control the position and shape of the distribution to reflect their views. The long top slider is for the expert to define the most likely value (the mode) and the two shorter sliders beneath separately control the uncertainty on each side of the mode (left standard deviation (sd) and right sd). The motivation for using this type of distribution is discussed below.

#### Quality of life scale:

The EQ-5D-5L is a generic measure of HrQoL which requires patients to describe their own health across five dimensions (mobility, self-care, usual activities, pain and discomfort, anxiety and depression) according to five levels. These responses are then combined with health state preference values from the UK general population, to give summary index scores on a scale anchored at 0 (death) and 1 (perfect health) (see Herdman et al. [15] for details). The multidimensional nature of the EQ-5D descriptive system raises additional challenges for eliciting expert opinion compared to disease-specific outcome measures that have one dimension (e.g. a pain scale). Our previous elicitation exercise attempted to address this problem by using the summary EQ-5D-5L index score. However, the initial piloting work for this elicitation exercise found that experts would find it easier to consider patients’ health status according to the individual dimensions (level of pain etc.). We therefore provided drop down menus for each of the five dimensions of health (Fig. 2, panel B, region d). All the levels are originally set to ‘moderate’ which gives an overall utility score of 62. The expert is then invited to see how the overall utility score changes according to the severity levels selected from the drop-down menu.

#### Representation of expert opinion:

The third of the challenges arises from the tension between wanting to provide experts with sufficient flexibility to accurately represent their beliefs, versus keeping the elicitation and analysis simple and transparent. In broad terms, there is a choice between parametric distributions and non-parametric distributions [19]. The parametric approach is considered more restrictive, while the non-parametric approach can be more unfamiliar, and hence more demanding for experts [19].

The crucial decision is the choice of distribution. Recall the principal focus of our elicitation is the mean score for the patients with missing outcome data. Even when the distribution of the individual scores is bimodal (e.g. the ‘too sick’ and the ‘too well’ do not respond), nevertheless an expert’s uncertainty about the *mean* will be reflected by a unimodal distribution. Hence, the flexibility of a non-parametric distribution is not required. However, using a normal distribution imposes symmetry, which is potentially inappropriate for bounded scales. Therefore, we propose using an asymmetric distribution controlled by three sliders (mode, left sd and right sd) as shown in Fig. 2. The underlying distribution is a (truncated) split normal distribution [20].

#### Define elicitation questions:

The structure, wording and use of feedback are central to preparing the elicitation tool [19]. Crucially, we must ensure the expert provides their views about the average score for a group of similar patients, rather than the scores for individual patients. To elicit enough information to specify the required priors requires outcomes to be elicited for patients who did not return their questionnaire for samples receiving usual care and the intervention, and it is also important to elicit values for the relationship between these two groups. These are described for the POPPI trial in the Supplementary material, and are easily adaptable.

For POPPI we elicited values for three patient subgroups according to patient characteristics that were anticipated to be potential effect modifiers. The three subgroups were A (female, younger and anxious after regaining capacity in the ICU), B (male, older and anxious) and C (male, younger and not anxious) — see Supplement for further detail. Free text questions asking the expert about the basis of their views can be included to provide useful context and facilitate the assessment of the expert’s responses to the quantitative questions.

#### Incorporate expert training:

The incorporation of expert training is dependent on how the elicitation will be conducted and the background of the experts. It is crucial to ensure the experts understand the tasks required of them and can correctly interpret the graphics displayed in the elicitation tool.

#### Pilot elicitation tool:

Piloting is a standard element of any elicitation exercise, and consists of an iterative process of refining the elicitation tool until we are confident that it can be used as intended.

### Step 3: elicit expert opinion

#### Recruit experts:

The aim should be to achieve a good coverage of all experts who understand what may predict whether or not patients respond to the questionnaire, and how outcomes may differ between those who do or do not respond to the questionnaire. It is also important to select experts who have some awareness of the potential effect that the intervention may have on outcomes, but also to select a range of experts rather than relying solely on those responsible for developing the intervention (see Supplement).

#### Conduct elicitation:

Options are in person (e.g. one-to-one interviews or in plenary sessions at conferences) or remote (on-line). If the latter is chosen (as for POPPI), the participants can be sent a link to the relevant questionnaire, along with the participant information sheet, and consent taken electronically. Where the elicitation tool is used remotely, the user can share their screen with the elicitation tool developer to facilitate completion. Reminders can be emailed (for POPPI these were sent at one week and four weeks).

### Step 4: evaluate elicitation results

#### Assessment of elicitation results:

In our application we tackled the fourth challenge by identifying those experts who provided ‘usable’ responses and within this sample we identified those experts in whom we had ‘high confidence’. This was done using pre-agreed principles; however – in a departure from our recommended approach – the detailed classification criteria were finalised after reviewing the elicited data. The ‘usable’ criteria excluded experts who had clearly misunderstood the task, evidenced by multiple unmoved sliders and their answers to the free text questions about the basis of their views. For the ‘high confidence’ group, we looked for 1) evidence of a high level of engagement with the elicitation exercise from qualitative answers; 2) consistency between their quantitative and qualitative answers and 3) consistency across the three subgroups. The elicited information was independently examined using these criteria by two of the authors (AM and DH), their categorisations compared, and discrepancies resolved through discussion. See the Supplementary material for examples.

#### Synthesise expert opinion

We advocate deriving pooled priors as the average of the individual distributions of a group of experts (e.g. all experts who provided ‘usable’ responses), based on linear pooling (on the distribution scale) with each expert weighted equally [19]. In the analysis these pooled priors are specified as a mixture of bivariate split normal distributions.

#### Select individual priors:

Individual priors based on the views of the ‘most sceptical’ expert and the ‘most enthusiastic’ expert can be chosen to reflect diversity of opinion. For the POPPI trial, we defined ‘most sceptical’ and ‘most enthusiastic’ to relate to the lowest and highest probability respectively that the mean outcome (PSS-SR/HrQoL) is better (lower/higher) for the POPPI intervention, and selected from the ‘high confidence’ group.

### Step 5: analyse trial data

#### Run statistical models

Examples from the POPPI trial are provided in the next section. The models were fitted using the WinBUGS software [21], and sample code is provided in the Supplementary material. For a more general discussion about the models, see Mason et al. [22].

#### Report results

This should include a discussion about the robustness of the base case results to the MNAR sensitivity analysis.

## Results

### Application to the POPPI trial

#### Expert elicitation

The 113 experts identified were randomly allocated to either of the PSS-SR or the HrQoL elicitation tools. Thirty-one from 57 experts completed the PSS-SR elicitation questionnaire, of whom 29 provided responses that were classified as ‘usable’ and of these 15 sets of responses met the criteria for ‘high confidence’. For the HrQoL version, 37 out of 56 responses were received (30 ‘usable’, of which 8 were ‘high confidence’). Table 1 shows the characteristics of the experts providing ‘usable’ responses.

**Table 1:** Summary of the characteristics of the experts who provided ‘usable’ responses

	PSS-SR		HrQoL		Total	
Number of ‘usable’ responses		29		30		59
of which, number of ‘high confidence’		15		8		23
Job: ^*a*^ n (% ^*c*^)						
Nurse	8	(28%)	5	(17%)	13	(22%)
Medical Doctor	16	(55%)	18	(62%)	34	(59%)
Other	5	(17%)	6	(21%)	11	(19%)
Years in current role: ^*a*^ n (% ^*c*^)						
1 year	1	(3%)	0	(0%)	1	(2%)
2 - 3 years	1	(3%)	1	(3%)	2	(3%)
4 - 6 years	3	(10%)	1	(3%)	4	(7%)
7 - 10 years	4	(14%)	4	(14%)	8	(14%)
more than 10 years	20	(69%)	23	(79%)	43	(74%)
POPPI site? ^*b*^ n (% ^*c*^)						
Yes (trial or feasibility)	8	(30%)	8	(29%)	16	(29%)
No	19	(70%)	20	(71%)	39	(71%)

^a^Excludes 1 HrQoL expert who did not respond to this question

^b^Excludes 1 HrQoL expert who did not respond to this question, and 2 PSS-SR experts and 1 HrQoL expert who were unsure

^c^Percentage of column total

**Fig. 3:**
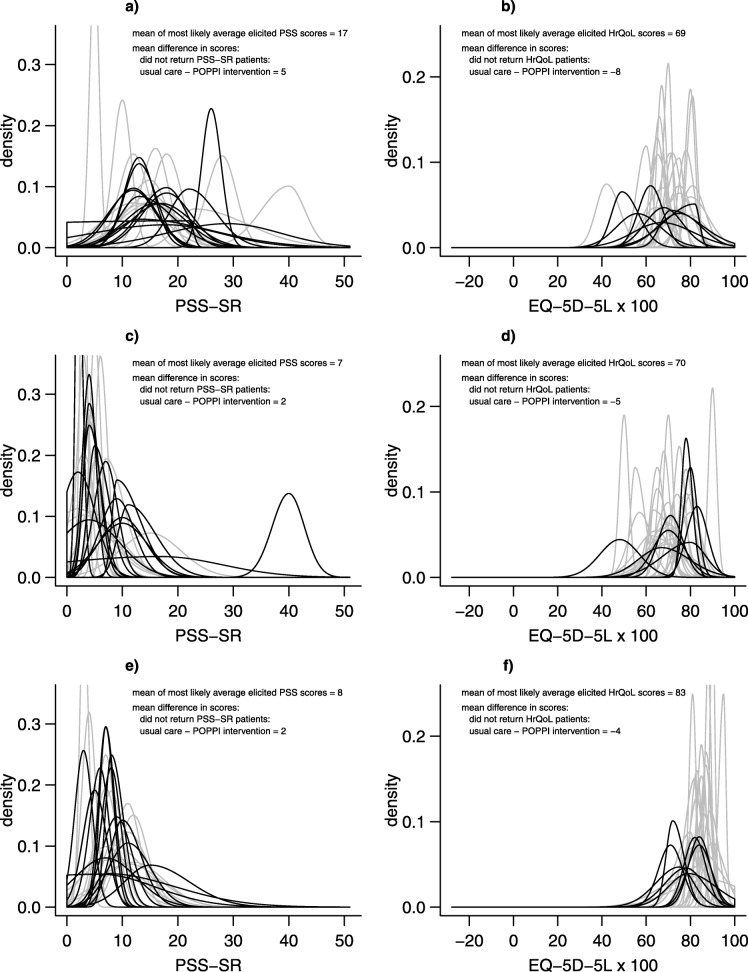
Individual elicited prior distributions for PSS-SR and HrQoL for patients receiving the POPPI intervention. Type A patients are female, younger and anxious; type B patients are male, older and anxious; type C patients are male, younger and not anxious. Thin grey lines = experts providing ‘usable but not with high confidence’ responses; thin black lines = experts providing ‘usable with high confidence’ responses. **a** PSS: Type A patients. **b** HrQoL: Type A patients. **c** PSS: Type B patients. **d** HrQoL: Type B patients. **e** PSS: Type C patients. **f** HrQoL: Type C patients

Figure 3 reports the diversity of views about the mean outcomes for those patients who received the POPPI intervention, but who did not complete the required questionnaires. The graphs in the left column show the distributions elicited for the average PSS-SR scores for patients who did not return their questionnaire for Type A (3a), B (3c) and C (3e) patients respectively, and the right hand panels for the same patient subgroups for the elicitation of HrQoL. Equivalent graphs for the patients who received usual care and an example of a joint prior can be found in the Supplementary material.

#### POPPI sensitivity analysis

**Fig. 4:**
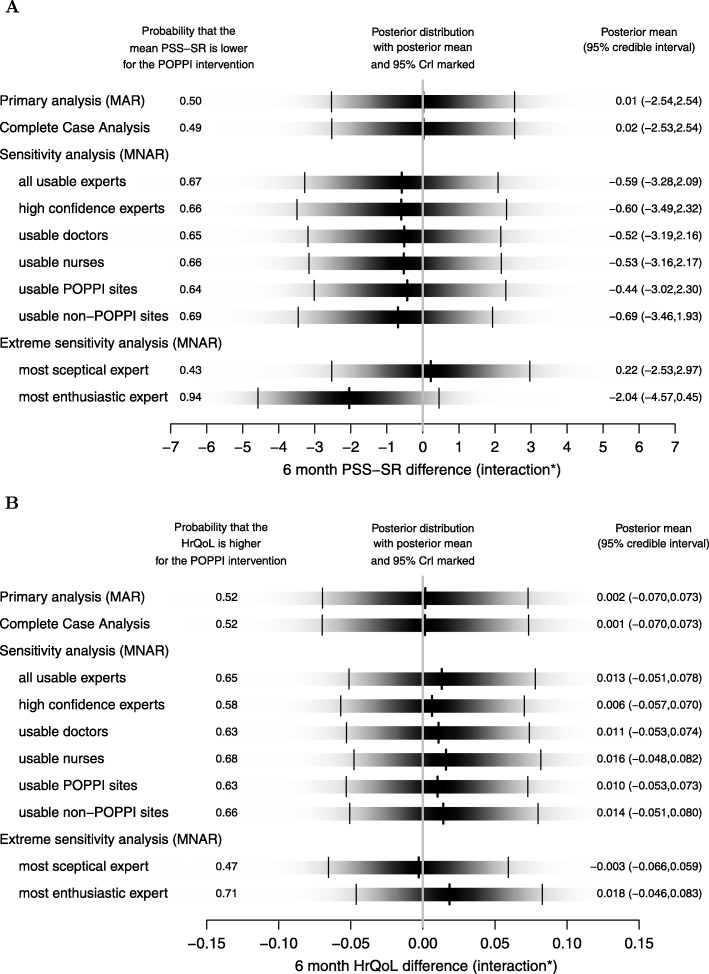
Treatment effect estimates at six months post-recruitment according to alternative missing not at random assumptions. The missing not at random assumptions are compared to the primary and complete case analyses. Each shaded rectangular strip shows the full posterior distribution. The darkness at a point is proportional to the probability density, such that the strip is darkest at the maximum density and fades into the background at the minimum density. The posterior mean and 95% credible interval (CrI) are marked. * interaction between treatment group and time period. For PSS-SR, negative differences favour the POPPI intervention, and for HrQoL positive differences favour the POPPI intervention. MAR = Missing At Random; MNAR = Missing Not At Random. **A** Primary treatment effect estimate. **B** Treatment effect on health-related quality of life score

The results of the sensitivity analysis compared to the primary analysis and complete case analysis can be interpreted from a Bayesian perspective and are summarised in Fig. 4. These show (1) the posterior probability that the outcome favours the POPPI intervention, and (2) the posterior distribution of the treatment effect (interaction between treatment group and time period). The full posterior distribution is shown as a density strip [23] where the darkness at a point is proportional to the probability density. The sensitivity analysis point estimates and associated uncertainty are generally similar to the primary analysis, and there are only slight differences between responses judged ‘usable’ and those meeting the ‘high confidence’ criteria. There are more substantial differences between the results of the extreme sensitivity analysis which is based on individual priors, with probabilities that the mean PSS-SR score is lower for the POPPI intervention of 43% and 94% for the ‘most sceptical’ expert and the ‘most enthusiastic’ expert respectively (for HrQoL extremes are 47% and 71%).

## Discussion

This paper develops a framework for eliciting the expert opinion required by missing data sensitivity analyses. This has been successfully applied to the POPPI trial, and the results included in the primary outcome paper [11]. We have used an ITT estimand, but the framework is applicable for other estimands; however we need to frame the questions appropriately for the estimand at hand. Our hope is that this will enable trialists to embed expert elicitation within their trial designs. Building on recent work [6, 8], we define the key steps required when eliciting expert views about those with missing versus observed outcome data. If the expert elicitation is embedded within the trial design, then the statistical analysis plan for the trial should include the appropriate missing data sensitivity analyses. In developing the framework, the paper has addressed four major challenges for expert elicitation that arise when eliciting values for sensitivity analyses.

First, experts with relevant knowledge must be identified, defined as those with information about the characteristics of the patients with missing outcome data, and their likely outcomes at follow-up. In the POPPI study, a total of 113 clinicians were judged to meet that criteria and were invited to undertake the elicitation exercise, of whom 68 (60%) completed the elicitation. Of these 59 experts provided responses which were judged ‘usable’. In future studies it could be helpful to extend the approach to expert identification to interview potential respondents: to identify the subset who already have relevant knowledge, provide training about the elicitation tasks, offer an opportunity for questions about the requirements of the task, and improve the feedback provided to the expert by the elicitation tool [19].

Second, the outcome scales must be presented to experts in a form that clearly relates to the patient’s health status. The POPPI trial provided the opportunity to illustrate how this challenge can be addressed with contrasting endpoints, a unidimensional disease-specific outcome (the PSS-SR) and a multidimensional HrQoL measure (the EQ-5D). The proportion of experts with ‘usable’ responses was higher for the PSS-SR, which was simpler and more familiar to the respondents. The elicitation for the EQ-5D was more demanding as it required responders to consider multidimensional aspects of the patients’ health. Nevertheless, the piloting work found that defining the EQ-5D according to the underlying health state descriptions was helpful, and improved upon the previous version developed for the IMPROVE trial [8]. Many health-technology assessments include the EQ-5D as an important secondary endpoint, and so customising this elicitation tool to help the expert understand and complete the task, will help future studies elicit accurate values for this measure.

Third, the POPPI study showed how expert opinion can be elicited to provide a realistic summary of the uncertainty around the crucial parameter, the mean value. Rather than assuming a symmetrical distribution as in previous elicitation exercises [8], we showed how ‘sliders’ could allow experts to report their uncertainty about the elicited values, recognising that an asymmetric distribution may better represent the experts’ views, especially when as with the EQ-5D, the scale is bounded.

Fourth, by carefully addressing the previous challenges raised in eliciting expert opinion, the study should be better placed to address the crucial concern that Heijtan and others have raised about the accuracy of the elicited versus the true values [9]. While the accuracy of the values elicited for the missing responses, is, by definition ‘unknowable’, strategies to address this concern are essential. Such strategies for evaluating the elicitation exercise should be embedded within the elicitation exercise. The approach taken in the POPPI study was for two reviewers to independently assess the elicitation results, drawing on the qualitative evidence provided by the experts. This information can be used to categorise responses that are ‘unusable’, ‘usable with low confidence’, and ‘usable with high confidence’. The results showed that the groups of respondents providing ‘high confidence’ responses reported greater uncertainty about the elicited mean values for the relatively complex elicitation tool (HrQoL), compared to the subsample who provided results that were ‘usable but with low confidence’. This provided a degree of reassurance on the likely accuracy of the elicited versus true values.

However, a promising alternative approach to ‘validation’ is to pose seed questions, whereby experts are asked questions where the truth is known [24, 25]. This provides a quantitative measure of the accuracy of each expert’s opinion, and could be used to weight each expert’s response to the main questions of interest. While such validation exercises have been undertaken elsewhere in the elicitation literature, developing appropriate seed questions - those similar to the primary research questions - is challenging in the context of trials and should be a high priority for future research.

The fully Bayesian approach to missing data, and associated sensitivity analysis, can be approximated using Multiple Imputation (MI). This may in principle be done using a joint model approach to MI [26], and has been implemented in the full conditional specification approach [27]. A popular alternative is the ‘tipping point’ approach, where we control the sensitivity analysis through a single parameter (either on the selection or pattern mixture scale), and move this parameter from the null value (typically corresponding to the primary analysis) until the conclusions change. The challenge is then to decide (typically post-hoc) how contextually plausible such tipping points are (see for example Mason et al. [22] and Carpenter and Kenward [28], Ch6 and references therein). For a more general overview of recent approaches, see the introduction to Cro et al. [29]. The attraction of our elicitation approach is that it seeks to quantify and capture the way experts instinctively adjust their interpretation of the trial analysis in the light of the proportion of missing data. The aim is that the results should reflect actual clinical experience, rather than be driven by statistical convenience.

We have illustrated our framework using a publicly funded, unblinded trial. The approach can also be applied for the primary analysis of blinded studies: we collect the expert opinion before unblinding, and then use it to perform sensitivity analysis to the primary analysis. Of course, this may not always be acceptable, as we may be concerned that conflicted experts could be biased toward a particular treatment. In such cases, the elicitation tool can be readily modified so that the questions do not require the expert to differentiate by randomised arm. In all studies, transparent reporting about the elicitation exercise and resulting priors is of paramount importance. In particular, if experts are required to postulate a treatment effect (which can be done before the trial is unblinded), then this requirement should be made clear in the reporting and interpreting of results.

## Conclusions

In conclusion, missing data raises particular challenges in clinical trials with Patient Reported Outcomes, because they may well be MNAR, and we need to explore the robustness of the trial’s conclusions to this possibility. Our proposed framework provides a series of practical steps for eliciting the information needed to inform rigorous missing data sensitivity analyses. We hope that this will give trialists the confidence to adopt this approach.

## Supplementary information

**Additional file 1** Includes information on:
• Further detail about the POPPI trial
• The elicitation questions for POPPI
• The POPPI subgroups
• Identification and recruitment of POPPI experts
• Assessment of elicitation results
• Derivation of joint expert priors
• Statistical models
• BUGS code
and 3 additional figures.

## Data Availability

The elicitation data generated and analysed during the current study are not publicly available but are available from the corresponding author on reasonable request.

## Abbreviations

HrQoL: Health-related quality of life
ICU: Intensive care unit
MAR: Missing at random
MI: Multiple imputation
MNAR: Missing not at random
POPPI: The psychological outcomes following a nurse-led preventive psychological intervention for critically ill patients trial
PROM: Patient-reported outcome measure
PSS-SR: PTSD symptom scale - self report questionnaire
RCT: Randomised controlled trial
SAP: Statistical analysis plan
sd: Standard deviation
