# Supplementary material for: A framework for extending trial design to facilitate missing data sensitivity analyses
Source: BMC Med Res Methodol. 2020 Mar 17;20:66. doi: 10.1186/s12874-020-00930-2 (PMC7076973; doi:10.1186/s12874-020-00930-2)
Supplement: Supplementary file 1 — Additional file 1 Includes information on: • Further detail about the POPPI trial • The elicitation questions for POPPI • The POPPI subgroups • Identification and recruitment of POPPI experts • Assessment of elicitation results • Derivation of joint expert priors • Statistical models • BUGS code and 3 additional figures. [file 12874_2020_930_MOESM1_ESM.pdf]

SUPPLEMENTARY MATERIAL

S1 Further detail about the POPPI trial

The POPPI intervention

The POPPI intervention was designed to alleviate acute stress and memories of frightening ICU experiences. It comprised three elements.

- Promotion of a therapeutic environment in ICU.
- Three stress support sessions for patients screened as acutely stressed, delivered by trained ICU nurses. These were commenced either in the ICU or following discharge to the hospital ward.
- A two-part relaxation and recovery programme for patients screened as acutely stressed. Part 1 was delivered during the patient's hospital stay, and part 2 was designed to give information on making a good psychological recovery after an ICU stay.

Wade *et al.* (2019) provide fuller information about the development and delivery of this intervention.

Randomisation

Randomisation was at the ICU level. Twenty-four ICUs were randomised, using a restricted approach to ensure balance, 1:1 to the intervention or control group using a stepped rollout. Intervention ICUs delivered usual care during a baseline period (months 1-5), followed by a 1-month transition period before delivering the intervention from month 7 onwards. Control ICUs delivered usual care throughout.

S2 The elicitation questions for POPPI

To start, we ask the expert to think about a group of 100 similar patients who were included in the trial, received usual care and returned a completed questionnaire. They are shown the outcome scale marked with our best estimate of the average score and associated uncertainty, based on a sample of this group of patients recruited early in the cluster-trial baseline period. Then, they are asked for their views about the average score for two more groups of 100 patients: I) similar to the original group, except they did not return their questionnaire and II) similar to the original group, except they received the POPPI intervention and did not return their questionnaire. At this stage they are provided with graphical feedback showing the difference/overlap in their views about the two groups (I and II) and asked to revise their answers if this seems unreasonable.

A key term in the statistical model is the correlation between the differences between observed and missing outcomes across intervention groups. This is a challenging parameter to elicit. To allow for the possibility that the elicited values for the missing usual care and

POPPI intervention patients are related, we also ask the expert to reconsider their views about the average score for the POPPI intervention patients in the light of new information about the usual care patients. Eliciting this third distribution provides sufficient information to formulate a joint distribution for the sensitivity parameters for each group allowing correlation between them. (See the section 'Derivation of joint expert priors' below for further detail.)

Because of the broad range of causes of ICU admission, the POPPI patient population is very heterogeneous. Consequently, we repeated these questions for three subgroups: A (female, younger and anxious after regaining capacity in the ICU), B (male, older and anxious) and C (male, younger and not anxious).

Free text questions asking the expert about the basis of their views were also included.

S3 The POPPI subgroups

Subgroup analysis was planned for the POPPI trial by age, gender, state trait anxiety inventory score (STAI) after regaining capacity in the ICU, socio-economic status, duration of delirium and surgical status. For practical reasons, we characterised subgroups for elicitation using only the first three listed factors with age (younger/older) and STAI (anxious/not anxious) dichotomised. These factors were chosen as they were considered most likely to be related to both response rates and longer term PTSD outcomes. However, eliciting for eight subgroups is still impractical, so we chose three taking into consideration response rates, number of non-respondents and the need to cover each option in the three binary splits. The chosen subgroups were: A (female, younger and anxious in unit), B (male, older and anxious in unit) and C (male, younger and not anxious in unit).

Individuals in the five non-elicited sub-groups were assigned a pre-specified elicited prior or mixture of elicited priors, assuming a priori that the observed differences in the distributions of the sub-groups would carry through to differences in the priors. The same allocation was reasonable for both outcomes, and is as follows:

- Female, younger and not anxious in unit - assigned prior C
- Female, older and not anxious in unit - assigned prior B
- Male, older and not anxious in unit - assigned prior B
- Male, younger and anxious in unit - assigned a mixture of priors A and C
- Female, older and anxious in unit - assigned a mixture of priors A and B.

S4 Identification and recruitment of POPPI experts

To identify participants to take part in the POPPI elicitation, the chief investigator sent a call for expressions of interest via email to the medical directors of all adult, general ICUs participating in the ICNARC Case Mix Programme and to Principal Investigators

of recent/ongoing ICU trials. The recipients were asked to identify the person most involved/interested in long-term follow-up of patients at their unit and to provide their contact details to ICNARC.

Those identified were then contacted via email, by the chief investigator, to confirm whether they would be interested in participating in the elicitation exercise. This approach resulted in a final sample of 113 individuals, of which 57 were randomly allocated to receive the PSS-SR elicitation tool, and the other 56, the HrQoL elicitation tool.

S5 Assessment of elicitation results

For POPPI, we categorised the expert responses into 'unusable', 'usable with low confidence' and 'usable with high confidence'. Here we provide examples for each category.

Example 1: unusable

The left standard deviation corresponded to the starting position of its slider for all the elicited distributions. The implication was that although the expert believed the average score could be higher than the value that they thought most likely, it could not be lower. As no rationale for this was provided in the qualitative answers, it was considered to be the result of not fully understanding how the sliders controlled the graph rather than a genuine view.

Example 2: unusable

In response to the free text questions about the reasons for their views, the expert commented:

> Would expect the scores to fall within that distribution.

We considered this to be evidence that the expert had misunderstood the elicitation tasks and provided a distribution relating to plausible individual patient scores instead of their uncertainty about an average score for a patient group.

Example 3: usable with low confidence

In their qualitative answers, the expert commented:

> Patients don't return questionnaires esp if feeling better. ... I think the intervention will have helped so scores will be higher.

Consistent with the final comment, their quantitative answers were higher for the POPPI intervention than usual care. However, for one patient subgroup these values were lower than for the corresponding observed value, which is inconsistent with the first comment. The expert may have had good reason for believing this subgroup is an exception, but no rationale is provided. Hence we decided that there was insufficient information in the qualitative answers to have high confidence in this expert's responses.

Example 4: usable with high confidence

This expert provided a detailed rationale for their views, starting as follows:

> Firstly, elderly patients are often more happy with their state of health following ICU discharge than younger patients; even if, objectively, they seem to have limitations to their functional status. Younger patients may have a different benchmark for what they consider to be a 'good' state of health, so in my experience often report more problems in the ICU clinic than the elderly; particularly if they are trying to return to work. Secondly, there are many factors which influence whether a patient will or will not return a questionnaire. If they are doing really well health-wise, they may decide not to attend clinic - we have certainly had several patients who have phoned to say they will not be attending as they have no problems, or even that they will be away overseas on holiday! However, it may be that poor health prevents patients from completing questionnaires. ...

They have clearly considered the different patient types and the reasons for not returning a questionnaire based on their experience. We decided that this demonstrates a high level of engagement with the elicitation exercise and that we can have high confidence in their quantitative responses.

S6 Derivation of joint expert priors

A joint prior for patients receiving usual care and patients receiving the POPPI intervention, based on the views of a single expert, is specified as a bivariate split normal distribution. This requires 7 parameters: 2 modes, 2 left sds, 2 right sds and a correlation. The values of the modes and sds are directly retrieved from the elicited marginal distributions.

To elicit the correlation, the expert is shown a slice through a bivariate split normal distribution in which the usual care difference is fixed (conditional distribution). This fixed usual care value is chosen using the experts's elicited marginal distribution for usual care patients (the expert is told that it is based on some hypothetical new scores). Specifically the 5th or 95th percentile of this distribution is selected according to furthest distance from the mode. Initially, the correlation in the bivariate distribution is assumed to be 0, so the required slice is the elicited marginal. However, this assumption can be modified by the expert, by moving a slider that is directly linked to the correlation parameter, to adjust this conditional distribution to better reflect their views (the range is restricted to ensure validity given the 6 parameters already elicited). If necessary the resulting bivariate split normal distribution is truncated to restrict the prior to legitimate values. For the PSS-SR elicitation, Figure S1 provides a contour line representation of an example of a joint prior based on the views of an expert whose responses were judged as 'high confidence'.

The truncated bivariate split normal distribution is not available in WinBUGS, so this was implemented by drawing from a random bivariate sample. This sample was generated externally from the elicited distribution and passed to WinBUGS as a data file. At each iteration, each member of this sample had equal probability of being picked. The random sample was created by inverse transform sampling using the R software.

S7 Statistical models

POPPI has a cluster RCT design, with 12 control sites and 12 intervention sites, each recruiting eligible admissions for 11 months. Control sites delivered usual care throughout. Intervention sites delivered usual care for 5 months, followed by a one month transition period and 5 months delivering the intervention. Hence, for patients who returned their completed questionnaire (pattern 1), the primary outcome (PSS-SR at 6 months) can be modelled using a Bayesian hierarchical model specified as follows:

$$\begin{aligned}
y_i &\sim \mathrm{N}(\mu_i, \sigma^2) \\
\mu_i &= \alpha_0 + \alpha_{s(i)} + \sum_j \beta_j x_{ij} + \sum_k \gamma_k z_{ik} \\
\alpha_s &\sim \mathrm{N}(0, \sigma_\alpha^2) \\
\alpha_0, \beta_j, \gamma_z &\sim \mathrm{N}(0, 1000^2) \\
\sigma^{-2} &\sim \mathrm{Gamma}(0.001, 0.001) \\
\sigma_\alpha &\sim \mathrm{Uniform}(0, 100)
\end{aligned}$$

where y_i is PSS-SR at 6 months for patient i, α_s is the random intercept for site s, $\boldsymbol{x}_i$ and $\boldsymbol{z}_i$ are vectors of patient level and site level fixed effects respectively for patient i. The patient level fixed effects include a treatment group (control or intervention site) $\times$ time period (months 1-5 or months 7-11) interaction term, and the treatment effect for patients with observed PSS-SR at 6 months is the associated β, which we denote by β_I. Patients recruited during the transition month are not modelled. This is the analysis model for pattern 1 of a pattern mixture model.

For the patients with missing scores (pattern 2), the equation for μ_i, includes an additional offset term δ_j, where $j = 1$ indicates the patient received usual care and $j = 2$ that they received the POPPI intervention (patients at an intervention site in the intervention time period). Values of δ_1 and δ_2 are drawn from the the elicited priors.

The mean treatment effect for patients with observed PSS-SR is β_I, and for patients with missing PSS-SR is $\delta_2 - \delta_1$ (δ_1 and δ_2 are both specified with reference to patients with observed PSS-SR receiving usual care). So, the overall treatment effect can be calculated as a weighted average of the treatment effect for patients with observed PSS-SR at 6 months and patients with missing PSS-SR at 6 months, i.e.

$$(\beta_I \times (1 - f)) + ((\delta_2 - \delta_1) \times f)$$

where f is the proportion of patients at POPPI sites receiving the POPPI intervention (patients recruited in months 7-11 at an intervention site) who have missing PSS-SR at 6 months. Negative numbers favour the intervention.

To allow different priors to be used for the subgroups, we introduce a second index into the offset term, δ_{jk}, where k indicates the prior (or combination of priors) to be used. The mean difference in the treatment effect for patients with missing PSS-SR at 6 months can then be calculated as a weighted average of the differences for each patient group, i.e.

$$\delta_A = \sum_k (\delta_{2k} - \delta_{1k}) \times wt_k$$

where wt_k is the proportion of the patients who received the POPPI intervention with missing scores for patient group k. For POPPI, 5 patient groups were defined: prior A, prior B, prior C, mixture prior A & B and mixture prior A & C (see discussion of POPPI subgroups above). The overall treatment effect can then be calculated as

$$(\beta_I \times (1 - f)) + (\delta_A \times f)$$

.

S8 Supplementary Figures

Figure S1: Example of the joint prior distributions for an individual expert providing 'usable with high confidence' responses

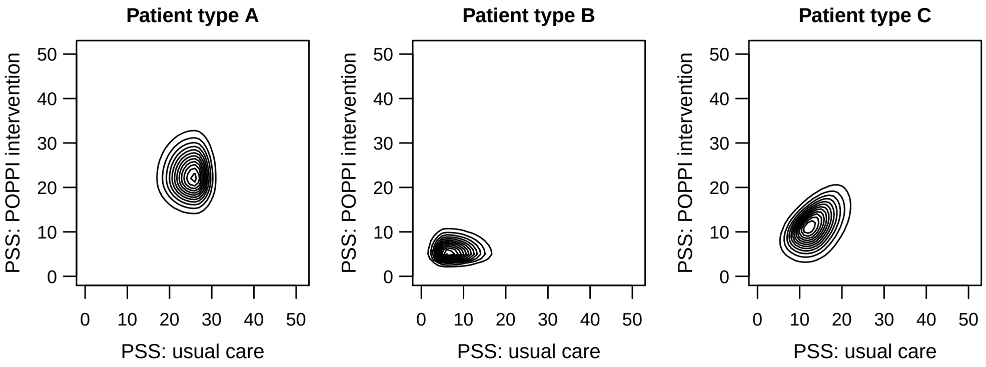

The black contour lines show the joint prior for an individual expert for the 3 types of patients elicited: A) female, younger and anxious; B) male, older and anxious and C) male, younger and not anxious.

Figure S2: Individual elicited prior distributions for PSS-SR and HrQoL for patients receiving usual care by patient type

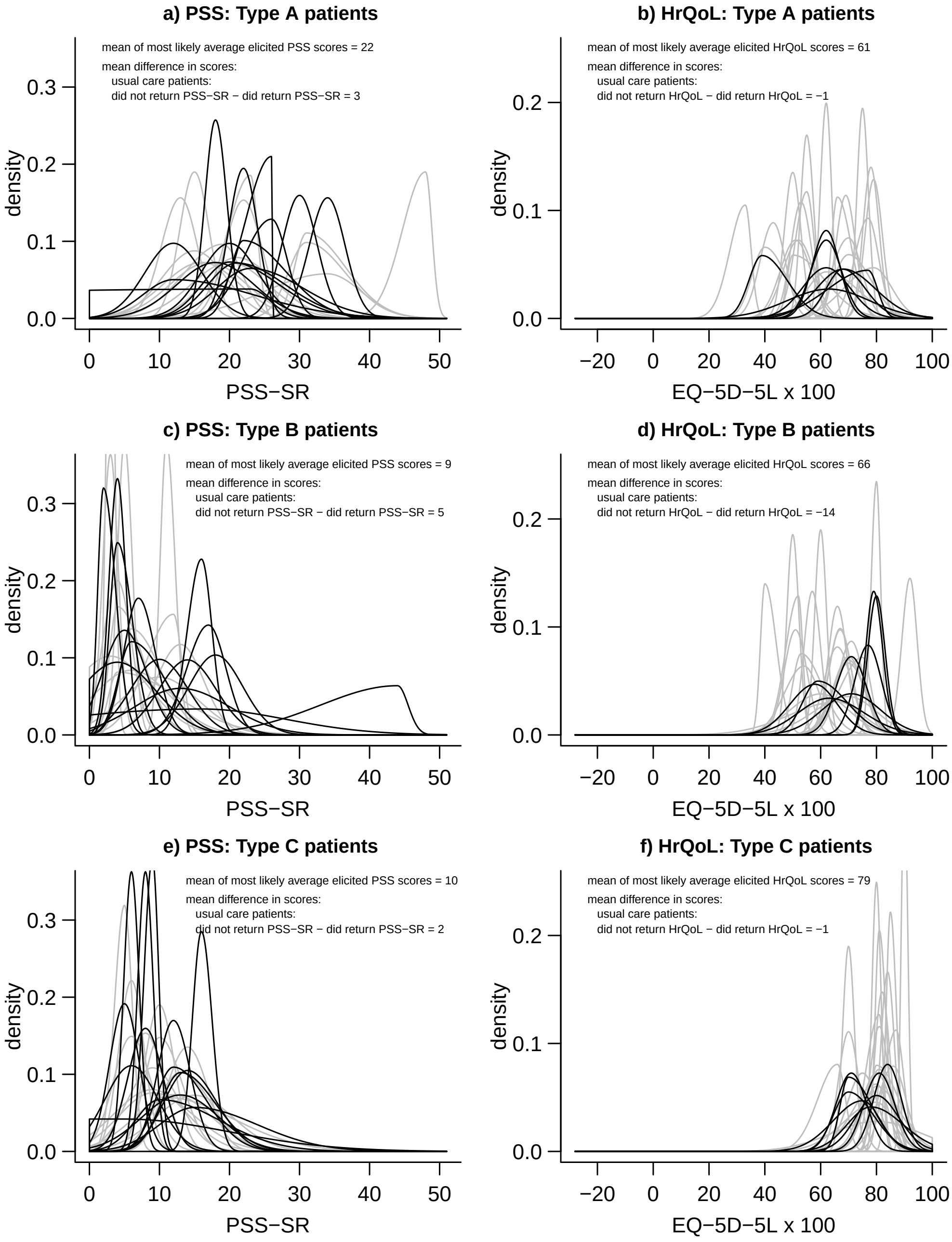

Type A patients are female, younger and anxious; type B patients are male, older and anxious; type C patients are male, younger and not anxious. Thin grey lines = experts providing ‘usable but not with high confidence’ responses; thin black lines = experts providing ‘usable with high confidence’ responses.

Figure S3: Incremental Net Benefit (valuing QALY gains at 20,000GBP per QALY) at six months post-recruitment according to alternative missing not at random assumptions compared to the primary and complete case analyses

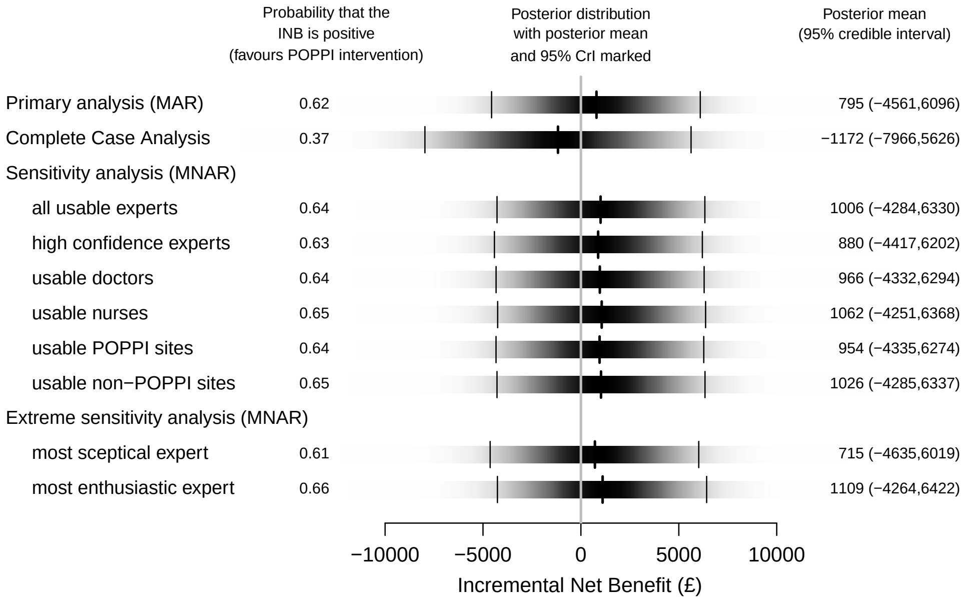

Each shaded rectangular strip shows the full posterior distribution. The darkness at a point is proportional to the probability density, such that the strip is darkest at the maximum density and fades into the background at the minimum density. The posterior mean and 95% credible interval (CrI) are marked.
The INB is calculated according to methods of the National Institute for Health and Care Excellence (NICE) by multiplying the mean gain or loss in quality-adjusted life-years by £20,000 and subtracting from this value the incremental cost.
MAR = Missing At Random; MNAR = Missing Not At Random

S9 BUGS code

```
# WinBUGS model code for Bayesian analysis of PSS-SR (PTSD symptom severity)
#   scores from the POPPI trial
# Outcome: PSS-SR at 6 months (pss)
# Pattern mixture model allows pss to be MNAR
# Uses pooled priors - a mixture of split normal distributions
# 5 patient types defined for selection of delta prior
#   1 = A; 2 = B; 3 = C; 4 = A/C; 5 = A/B (3 elicited subgroups + 2 combinations)
# Site random intercepts - not hierarchically centred
# Fixed effects at site level:
#   teaching status of hospital (ts: non-teaching=0, teaching=1)
#   number of beds in the critical care unit - centered (nbs)
#   number of critical care unit admissions receiving level 3 care - centered (nl3)
#   allocated treatment group (trt: control arm=0, treatment arm=1)
# Fixed effects at patient level:
#   time period (tp: baseline=0, intervention=1)
#   interaction between time period and treatment group
#   age in years - centered (cage): cubic spline with 4 knots
#   gender (sex: male=0, female=1)
#   ethnicity (eth: white=0, non-white=1, not reported=2)
#   quintile of IMD 2015 (imd: 5 categories)
#   pre-existing anxiety and/or depression (anx: none=1, anxiety=2, depression=3, both=4)
#   admission type (adm: no=0, yes=1)
#   ICNARC Physiology Score - centered (cps): cubic spline with 4 knots

model{
  for (i in 1:N) { # individuals
    pss[i] ~ dnorm(mu[i],tau)
    # Last term adjusts for patients with missing pss who received poppi
    #   intevention, because offset (delta) is w.r.t. patient with observed
    #   pss receiving usual care
    mu[i] <- alpha0 + alpha[site[i]] + pat.fe[i] + site.fe[i] +
             delta[grp[i],ptype[i]] * miss[i] -
             (beta.int + gamma[4]) * miss[i] * (grp[i] - 1)
    # Fixed effects at site level
    site.fe[i] <- gamma[1]*ts[i] + gamma[2]*nbs[i] + gamma[3]*nl3[i] +
                  gamma[4]*trt[i]
    # Fixed effects at patient level
    pat.fe[i] <- beta.tp*tp[i] + beta.int*tp[i]*trt[i] + age.rcs[i] +
                 beta.sex*sex[i] + beta.eth[eth[i]] + beta.imd[imd[i]] +
                 beta.anx[anx[i]] + beta.adm*adm[i] + ps.rcs[i]
    # Restricted cubic spline for age
    age.rcs[i] <- beta.age[1]*cage[i] + beta.age[2]*cage2[i] + beta.age[3]*cage3[i]
    # Restricted cubic spline for ICNARC Physiology Score
    ps.rcs[i] <- beta.ps[1]*cps[i] + beta.ps[2]*cps2[i] + beta.ps[3]*cps3[i]
    grp[i] <- (trt[i] * tp[i]) + 1 # 1 = usual care, 2 = poppi intervention (patients
                                   # at intervention site in intervention time period)
  }
```

```
for (s in 1:Nsite) { alpha[s] ~ dnorm(0,alpha.tau) } # random intercepts

icc <- alpha.sigma2/(alpha.sigma2+sigma2)

# Priors
alpha0 ~ dnorm(0,1.0E-6)
beta.tp ~ dnorm(0,1.0E-6)
beta.int ~ dnorm(0,1.0E-6)
for (i in 1:3) {beta.age[i] ~ dnorm(0,1.0E-6)}
beta.sex ~ dnorm(0,1.0E-6)
beta.eth[1] <- 0
for (i in 2:3) {beta.eth[i] ~ dnorm(0,1.0E-6)}
beta.imd[1] <- 0
for (i in 2:5) {beta.imd[i] ~ dnorm(0,1.0E-6)}
beta.anx[1] <- 0
for (i in 2:4) {beta.anx[i] ~ dnorm(0,1.0E-6)}
beta.adm ~ dnorm(0,1.0E-6)
for (i in 1:3) {beta.ps[i] ~ dnorm(0,1.0E-6)}
for (i in 1:4) {gamma[i] ~ dnorm(0,1.0E-6)}
tau ~ dgamma(0.001,0.001)
sigma2 <- 1/tau
sigma <- sqrt(sigma2)

# Priors for random effects
alpha.sigma ~ dunif(0,100)
alpha.sigma2 <- pow(alpha.sigma,2)
alpha.tau <- 1/alpha.sigma2

# Priors for score differences - a mixture of split normal distributions
pick ~ dcat(Q[]) # pick element (data point) from sample from prior distributions
for (i in 1:Ndpts) {Q[i] <- 1/Ndpts}
pick.e ~ dcat(S[]) # pick expert
for (i in 1:Nexperts) {S[i] <- 1/Nexperts}
delta[1,1] <- dpriorA[pick,1,pick.e]
delta[2,1] <- dpriorA[pick,2,pick.e]
delta[1,2] <- dpriorB[pick,1,pick.e]
delta[2,2] <- dpriorB[pick,2,pick.e]
delta[1,3] <- dpriorC[pick,1,pick.e]
delta[2,3] <- dpriorC[pick,2,pick.e]
pick.sg ~ dcat(R[]) # pick subgroup where choice of two
for (i in 1:2) {R[i] <- 1/2}
sg <- pick.sg-1
delta[1,4] <- ((1-sg)*delta[1,1] ) + (sg*delta[1,3]) # A if sg=0; C if sg=1
delta[2,4] <-  ((1-sg)*delta[2,1] ) + (sg*delta[2,3]) # A if sg=0; C if sg=1
delta[1,5] <- ((1-sg)*delta[1,1] ) + (sg*delta[1,2]) # A if sg=0; B if sg=1
delta[2,5] <- ((1-sg)*delta[2,1] ) + (sg*delta[2,2]) # A if sg=0; B if sg=1
for (i in 1:5) {delta.diff[i] <- delta[2,i]-delta[1,i]}
ave.delta.diff <- inprod(delta.diff[],ptype.wts[])
```

```
  # Calculate pss difference: poppi intervention - usual care
  # Negative numbers favour intervention
  # miss.frac is the proportion of patients at poppi sites receiving the poppi
  #   intervention (trt=1 and tp=1) who are missing
  pss.diff <- beta.int * (1-miss.frac)  +  ave.delta.diff * miss.frac

  # Probability intervention reduces pss score
  psuccess.obs <- 1-step(beta.int)
  psuccess.mis <- 1-step(ave.delta.diff)
  psuccess <- 1-step(pss.diff)
}
```

References

Wade, D. M., Mouncey, P. R., Richards-Belle, A., Wulff, J., Harrison, D. A., Sadique, Z., Grieve, R. D., Emerson, L. M., Mason, A. J., Aaronovitch, D., Als, N., Brewin, C. R., Harvey, S. E., Howell, D. C. J., Hudson, N., Mythen, M. G., Smyth, D., Weinman, J., Welch, J., Whitman, C., and Rowan, K. M. (2019). Effect of a Nurse-Led Preventive Psychological Intervention on Symptoms of Posttraumatic Stress Disorder Among Critically Ill Patients: A Randomized Clinical Trial. *JAMA*, **321**, (7), 665–75.
